# The mitochondria as an emerging target of self-renewal in T-cell acute lymphoblastic leukemia

**DOI:** 10.1080/15384047.2025.2460252

**Published:** 2025-02-04

**Authors:** Majd A. Al-Hamaly, Evelyn Winter, Jessica S. Blackburn

**Affiliations:** a Pharmacology and Nutritional Sciences, University of Kentucky, Lexington, KY, USA; b Markey Cancer Center, University of Kentucky, Lexington, KY, USA; c Department of Agriculture, Biodiversity and Forestry, Federal University of Santa Catarina, Curitibanos, Brazil; d Molecular and Cellular Biochemistry, University of Kentucky, Lexington, KY, USA

**Keywords:** Mitochondria, OXPHOS, metabolism, self-renewal, leukemia stem cells, cancer stem cells, T-ALL

## Abstract

Acute lymphocytic leukemia (ALL) is the most common leukemia in children, with the T-cell subtype (T-ALL) accounting for 15% of those cases. Despite advancements in the treatment of T-ALL, patients still face a dismal prognosis following their first relapse. Relapse can be attributed to the inability of chemotherapy agents to eradicate leukemia stem cells (LSC), which possess self-renewal capabilities and are responsible for the long-term maintenance of the disease. Mitochondria have been recognized as a therapeutic vulnerability for cancer stem cells, including LSCs. Mitocans have shown promise in T-ALL both *in vitro* and *in vivo*, with some currently in early-phase clinical trials. However, due to challenges in studying LSCs in T-ALL, our understanding of how mitochondrial function influences self-renewal remains limited. This review highlights the emerging literature on targeting mitochondria in diverse T-ALL models, emphasizing specific mitochondrial vulnerabilities linked to LSC self-renewal and their potential to significantly improve T-ALL treatment.

## 1. Introduction

Leukemia is the most prevalent pediatric cancer, representing one-third of all childhood cancers. Acute lymphocytic leukemia (ALL) accounting for more than 80% of those cases.^1^ T-cell acute lymphoblastic leukemia (T-ALL), characterized by the malignant transformation of T-lymphocytes precursor cells, represents 10–15% of pediatric ALL.^2^

The treatment of ALL has seen significant advancements; a once fatal cancer with a survival rate of only 11% in the 1960s^3^ now boasts a 90% cure rate for B-cell ALL (B-ALL).^4^ However, cure rates of T-ALL patients remain lower, at approximately 72%.^4,5^ Following the first relapse, T-ALL patients often develop multi-drug resistance and become refractory to second-line treatments.^6^ These patients face a grim outlook. Relapsed T-ALL patients have a 5-year overall survival (OS) rate of 20–40%,^7^ with a median OS of just 4–5 months.^8^

Relapse in leukemia is often attributed to the failure of cytotoxic chemotherapy agents to eradicate leukemia stem cells (LSCs), which may be quiescent or have inherent drug-resistance mechanisms.^9^ The cancer stem cell (CSC) hypothesis posits that a small subset of cells within a tumor possesses an indefinite ability to self-renew, is responsible for the long-term maintenance of tumor growth, and can potentially repopulate the cancer from just a single cell.^10^ Research into LSCs in T-ALL has advanced significantly. Leukemia-initiating activity being identified in a distinct subpopulation of cells in patient samples,^11^ in T-ALL mouse^12^ and zebrafish models.^13^ These cells can induce leukemia after transplantation to a secondary host, retain the immunophenotypic characteristics of the primary leukemia, and maintain the ability to self-renew after serial transplantation.^14,15^ Consequently, the eradication of LSCs holds great promise to achieve completed disease regression and improved clinical outcomes for T-ALL.^16^

Currently, no approved therapies are specifically designed to target the self-renewal processes of leukemia stem cells in T-ALL. Existing therapeutic strategies under development for T-ALL predominantly focus on the modulation of dysregulated signaling pathways, including Notch, mTOR, and PI3K.^17^ However, these pathways are also important in the development and function of the normal hematopoietic system, and their utilization to target LSCs has resulted in side effects limiting their clinical applications.^18^ Hence, there is a need for innovative therapeutic approaches to effectively target LSCs in T-ALL with an enhanced safety profile and increased potential for a successful transition to clinical development.

The role of the mitochondria conferring stemness to cancer stem cells^19^ and their contribution to drug resistance^20^ has been increasingly recognized. This positions mitochondrial function as a pivotal target for therapies aimed at eliminating CSCs.^21^ The mitochondria in CSCs exhibit distinct characteristics compared to those in the rest of the tumor. Those unique features may play a central role in maintaining stemness and regulating cell proliferation and apoptosis.^19^ Such features include mitochondrial morphology,^22^ subcellular localization,^23^ the amount of mitochondrial DNA (mtDNA)^24^ and mitochondrial metabolism.^25^ These characteristics have been extensively studied in CSCs and compared to their differentiated counterparts in various cancers, such as liver,^26^ breast^27^ and oral^28^ cancers.

In T-ALL, targeting the mitochondria is emerging as a promising strategy for anti-leukemia therapies, either as a standalone treatment or in combination with conventional chemotherapy agents.^29^ However, investigating mitochondrial alterations specifically associated with stemness in T-ALL presents challenges, primarily due to the lack of definitive surface markers for LSCs, necessitating functional assays to interrogate self-renewal.^30^ Despite these obstacles, the specific contributions of mitochondria to LSC self-renewal are becoming more apparent. This review will explore the current understanding and emerging insights into the role of mitochondria in self-renewal, as well as the potential mitochondrial vulnerabilities of LSCs.

## 2. T-ALL: epidemiology and current landscape

### 2.1. Genetic insights and treatment approaches

T-ALL accounts for 10–15% of pediatric ALL cases, with an incidence 2–3 times higher in boys than in girls. These patients often present with high initial white blood cell count, a higher frequency of mediastinal mass, and neurological abnormalities.^31^ Genetic and epigenetic abnormalities in immature thymocytes can lead to T-ALL or T-cell lymphoblastic lymphoma (T-LBL).^1,32^ Predominantly, three pathways are dysregulated in T-ALL. First, over 90% of T-ALL cases can be classified into subgroups based on the deregulation of T-ALL transcription factors such as *TAL1, TAL2*, and *LMO2/LYL1*. Second, the aberrant activation of *NOTCH1* signaling, either through activating mutations (in more than 75% of cases) or mutations that inhibit the negative regulator *FBXW7*, leads to high Myc expression and uncontrolled cell proliferation. Finally, there is often a deletion of the tumor suppressor genes *CDKN2A/CDKN2B*.^33^

Treatment for newly diagnosed T-ALL patients involves intensive chemotherapy structured in three phases: induction, consolidation, and maintenance. The goal of induction therapy is to restore normal blood cell levels, and it typically includes vincristine, corticosteroids, and asparaginase with or without anthracycline for 4–6 weeks. Following that, consolidation therapy aims to eliminate the residual leukemia cells, which is achieved using a combination of high-dose chemotherapy agents, including methotrexate. Maintenance therapy lasts for 1–2 years and includes 6-mercaptopurine (6-MP) and methotrexate to prevent relapse.^34^ Hematopoietic stem cell transplantation is reserved for adult T-ALL patients at high risk for relapse.^35^

### 2.2. Addressing treatment challenges in T-ALL

Pediatric patients exhibit unique drug absorption and metabolic profiles compared to adults, making toxicity a crucial consideration in intensive chemotherapy protocols.^36^ Despite high cure rates, treatment-related morbidity and adverse event rates in pediatric T-ALL remain significant. Summers et al. evaluated 120 children aged 1–21 with T-ALL to assess clinically relevant adverse effects (AEs) resulting from chemotherapy. The majority of patients, 70% during induction and 80% during consolidation, experienced AEs.^37^ The chemotherapy-induced gonadotoxic effects of chemotherapy pose another significant concern. Close et al. categorized different leukemia treatment protocols according to the risk they pose for gonadal dysfunction/infertility. More than half (54%) of the ALL treatment protocols present a high risk, particularly for male patients, who comprise the higher proportion of the affected demographic.^38^

The emergence of Chimeric Antigen Receptors (CAR)-T cell therapy (CAR T-cell) marked a significant breakthrough in the treatment of hematological malignancies, offering a possible solution for the challenges associated with conventional chemotherapies.^39^ CAR-T cells are engineered to identify antigens presented by cancer cells and initiate a potent anti-tumor response. In B-ALL, the US Food and Drug Administration (FDA) approval of two CD19-targeted CAR-T cell therapies has been particularly transformative for patients refractory to initial chemotherapy or experiencing relapse,^40^ with phase II clinical trials in pediatric and adolescent patients reporting an 81% remission rate at day 28 post-infusion. These high remission rates have been confirmed by real-world data.^41^ However, despite the promise of CAR-T cell therapies, they are associated with significant limitations, including severe side effects such as cytokine release syndrome and neurotoxicity.^42^ For instance, 23–46% of patients treated with CAR-T therapies experienced supraphysiological cytokine production, leading to massive T-cell expansion, Macrophage Activation Syndrome, renal failure, pulmonary edema, and immune effector cell-associated neurotoxicity.^43^ Additionally, the high cost of FDA-approved CAR T-cell therapies raises serious concerns about feasibility and affordability for the general patient population.^44^ Finally, no approved CAR-T is available for standard clinical care for T-ALL patients. CD7 CAR-T cell therapy is currently under investigation for T-ALL, with ongoing clinical trials evaluating its safety and efficacy in patients with CD7-positive malignancies.^45–47^ This gap in available treatments highlights the urgent need for continued research and development of effective therapies for T-ALL.

### 2.3. Prognostic indicators and relapse challenges

The most pressing challenge in the treatment of T-ALL across pediatric and adult populations is the occurrence of relapse.^48^ Minimal residual disease (MRD), defined as the persistence of cancer cells post-treatment, is the most important prognostic factor for assessing relapse risks.^49^ MRD levels are typically assessed by flow cytometry, which has a detection threshold of 0.01%.^50^ More recently, clonoSEQ has been approved as a next-generation sequencing (NGS) platform for MRD measurement in ALL.^51^ This assay utilizes multiplex PCR and NGS techniques to identify leukemia-specific B or T cell receptor gene rearrangements.^52^ The NGS-based MRD testing boasts a high sensitivity, estimated at 10^−7.53^ Momen et al. reported that MRD detected exclusively by clonoSEQ but not by flow cytometry corresponded to lower MRD values.^54^ Based on the MRD scores at specific time points, patients are stratified into standard, intermediate, and high-risk groups, enhancing the predictive accuracy of patient outcomes.^34^ The presence of MRD is now considered the strongest predictor of patient relapse.^55^ Yet, the limited tolerability of cytotoxic chemotherapies makes achieving remission after a relapse difficult, often leading to poor outcomes. For instance, in the E2993/UKALL12 study, only 8 out of 123 adult T-ALL patients who relapsed survived after a median follow-up of 5.2 years.^56^ A similar retrospective analysis of relapsed pediatric T-ALL patients showed a 10-year overall survival rate ranging from 13% to 29%,^57^ highlighting the grim prognosis for patients post-relapse.

In total, the field of pediatric T-ALL treatment faces three critical challenges: the morbidities associated with chemotherapy, the limitations of still-developing CAR T-cell therapies for T-cell malignancies, and the bleak prognosis for patients who relapse. These issues highlight a significant gap in effective treatment strategies. To overcome these challenges, there is a pressing need for innovative therapies that target the biological underpinnings of the disease.

## 3. Targeting leukemia stem cells for improved clinical outcomes

**Figure 1.**
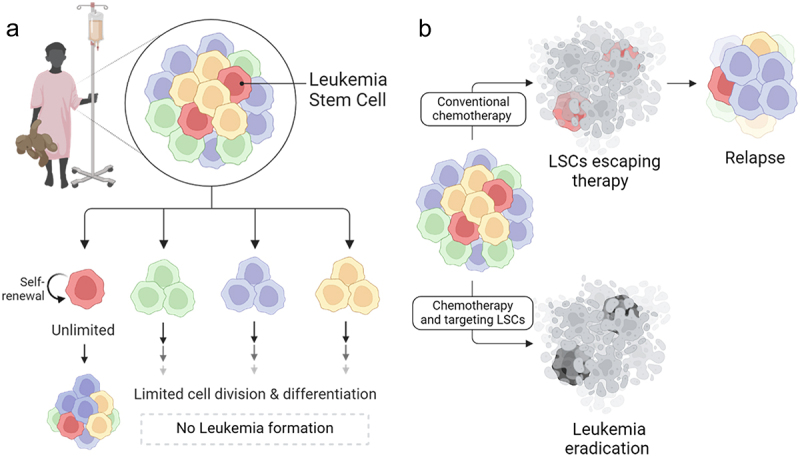
Leukemia stem cells are responsible for the long-term maintenance of the disease and patient relapse. a: Leukemia stem cells are a subset of cells within T-ALL that have the capacity to self-renew and regenerate disease from low cell number, compared to the rest of the population that have limited cell divisions. b: Conventional chemotherapy drugs are targeted toward the rapidly proliferating leukemia cells and may not always eradicate LSCs, resulting in patient relapse. Treatment strategies that combine LSC targeting therapies with conventional chemotherapy can eradicate the entire disease and prevent recurrence.

Intensive, cytotoxic chemotherapy remains the standard of care in treating T-ALL and effectively eliminates proliferating cells. However, these therapies often target the bulk of leukemia cells indiscriminately, which can result in a dramatic initial response but may fail to induce a complete and long-term remission.^58^ This outcome is particularly evident in patients with high MRD values, who are at greatest risk of relapse. The ability of MRD cells to reform leukemia from low cell numbers can be understood through the cancer stem cell model. In T-ALL, this model highlights leukemia stem cells (LSCs) as a critical subpopulation.^59^ These cells possess an indefinite capacity for self-renew and are essential for sustaining long-term tumor growth.^60^ Importantly, only the LSC subset can reestablish leukemia, while the other, more differentiated sub-populations have a limited capacity for long-term self-renewal and proliferation (Figure 1a).^10^ Targeting and eliminating these LSCs is essential for patients to achieve durable remission (Figure 1b).^59^

### 3.1. Challenges in defining leukemia stem cell markers in T-ALL

A particular challenge in targeting T-ALL self-renewal involves the lack of robust surface markers for LSCs in this disease. Cox et al. utilized pediatric leukemia patient samples in engraftment studies with NOD/SCID mice and demonstrated the ability of CD34+/CD4− and CD34+/CD7− subfractions to populate leukemia after serial passaging.^61^ In contrast, Gerby et al. reported that the CD34+/CD7- subpopulation isolated from the peripheral blood of T-ALL patients contained normal human progenitors that underwent normal hematopoietic differentiation *in vitro* and *in vivo*. Instead, they described the enrichment of LSCs in the CD34+/CD7+ fraction.^11^ In addition, Chiu et al. showed that a subset of cells that are CD7+CD1a− from primary T-ALL patient samples are enriched for LSC by xenograft studies in immune-deficient mice and noted CD34 as a reliable stemness marker for some but not all of the samples tested.^62^ Advanced single-cell sequencing technologies have also revealed significant heterogeneity within the LSC population in T-ALL. Zhu et al. analysis used single-cell transcriptomics and showed that cells expressing high levels of TIM-3/HAVCR2 were enriched for pathways associated with hematopoietic stem cells, late hematopoietic progenitors, and cellular quiescence. These cells also demonstrated the highest leukemia-initiating capacity, confirmed by a limiting dilution assay in immune-deficient mice. Almost every cell in this high TIM-3/HAVCR2 expression group was able to induce T-ALL development compared to 1 in 28 in the low-expression group.^63^ In a related study, Panelli et al. reported that CD117 and CD82 characterized a distinct T-ALL subpopulation enriched in MRD. This observation was further validated by injecting CD117+CD82+ T-ALL cells into immune-deficient mice at limiting dilution and finding enrichment in the leukemia-initiating capacity in this population of cells in two independent primary T-ALL samples.^64^ Taken together, these findings imply that the LSC activity in T-ALL might originate from different subpopulations of cells and underscore the heterogeneity within LSCs. Until cell surface makers can be reliably defined for LSCs in T-ALL, there is increasing dependence on functional methodologies to assess LSC self-renewal capabilities. Techniques such as the limiting dilution transplantation studies in immune-deficient mice are widely used,^65,66^ and more recently, similar studies have been conducted in syngeneic clonal zebrafish models.^67,68^ Continued research is needed to better understand LSC distribution in T-ALL and to validate newly described surface markers across different T-ALL animal models and patient samples. Ultimately, the identification of robust LSC surface makers will be critical for advancing self-renewal-focused drug screens and the discovery of novel therapeutic targets for T-ALL.

### 3.2. Mechanisms of leukemia stem cell survival and drug resistance

Intriguingly, the prevalence of LSCs within the leukemia population is closely linked to disease pathogenesis. Ho et al. monitored a cohort of acute myelogenous leukemia (AML) patients using limiting dilution analysis to quantity LSC frequency. They observed a dramatic 9- to 90-fold increase in LSC frequency between diagnosis and relapse,^69^ underscoring the critical role LSCs play in disease progression and recurrence. Another study found that the presence of LSCs in AML, identified with a CD34+/CD38− immune phenotype, strongly predicted patient outcomes.^70^ Patients with higher LSC frequencies (>3.5%) had a median relapse-free survival of 5.6 months compared to 16 months for those with lower frequencies of these cells,^71^ highlighting the significant impact of LSC burden on clinical prognosis.

**Figure 2.**
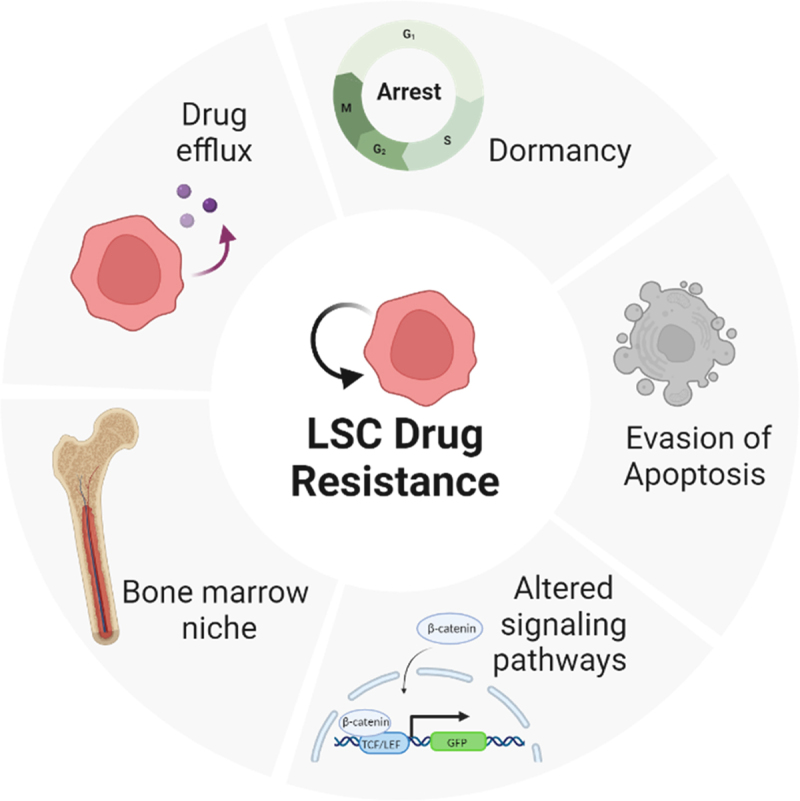
LSCs have unique mechanisms in place to overcome chemotherapy. Leukemia stem cells develop multiple mechanisms to escape conventional chemotherapy treatment including the expression of drugs efflux pumps, cell cycle arrest, their interaction with the bone marrow micromovement and the altered signaling and apoptosis regulation pathways.

The relationship between LSC prevalence and patient prognosis is evident. A deeper understanding of LSC biology is crucial to addressing their role in disease progression and treatment resistance, particularly their mechanisms of chemoresistance and survival. Work in AML, where LSCs have been well-characterized, shows that this cell type possesses several unique properties that allow it to evade conventional cytotoxic chemotherapy (Figure 2).^72^ Notably, LSCs have higher expression of ATP-binding cassette (ABC) transporters, which are key players in drug resistance. These include the permeability glycoprotein (P-gp), breast cancer resistance protein (BCRP), and multidrug resistance-associated protein 1 (MRP1).^73^ Additionally, LSCs often exist in a state of dormancy or slowed growth, allowing them to circumvent the effects of antiproliferative chemotherapy agents.^74^ In T-ALL patient-derived xenograft (PDX) mouse models, cells demonstrating the slowest growth rate and highest retention of the cell proliferation stain CFSE were associated with stemness, drug resistance, and high similarity to MRD cells.^75^ LSCs maintain dormancy by activating pathways central to self-renewal and stemness, such as the Wnt/β-catenin signaling pathway. In T-ALL patients, about 85%of the patients have an upregulation in the expression of β-Catenin and subsequent high expression of Wnt target genes, including c-MYC, TCF, and LEF.^76^ Single-cell sequencing of MRD samples in T-ALL patients also identified high expression of β-Catenin in this drug-resistant cell population.^77^ LSCs also display defects in apoptosis-related signaling pathways, such as the upregulation of the anti-apoptotic protein BCL-2.^78^

While these intrinsic mechanisms are pivotal, the tumor microenvironment (TME) also plays an essential role in maintaining LSC survival and drug resistance. In normal tissues, niches provide essential cues to regulate stem cell differentiation and quiescence,^79^ and in cancer, these niches support cancer stem cells by promoting self-renewal and recruiting stromal and immune cells to drive tumor progression.^80^ In leukemia, the bone marrow niche, which tightly regulates hematopoietic stem cells (HSCs),^81^ is co-opted by LSCs to gain survival advantages and evade therapy.^82^ This niche-driven support includes mitochondrial transfer from stromal cells to LSCs,^83^ cytokine-mediated signaling,^84^ and the release of extracellular vesicles,^85^ which collectively modulates drug resistance pathways. By leveraging these niche-specific interactions, LSCs evade therapeutic pressures, contributing to MRD and increasing the likelihood of relapse.

### 3.3. Therapeutic strategies targeting leukemia stem cells

The intrinsic survival mechanisms and niche-driven support that sustain leukemia stem cells (LSCs) highlight the challenges of effectively targeting these cells. Building on this understanding, researchers have developed therapeutic strategies aimed at disrupting LSC survival and self-renewal pathways. Notable success has been achieved with all-trans retinoic acid (ATRA) in the treatment of acute promyelocytic leukemia (APL). ATRA induces terminal differentiation of APL blasts into functional granulocytes.^86^ This approach has successfully been translated into clinical settings; patients receiving a combination therapy of ATRA and arsenic trioxide (ATO) showed superior outcomes compared to those on ATO monotherapy. All patients (*n* = 20) treated with this combination remained in remission, while 19% (*n* = 37) of those receiving only ATO relapsed within a median follow-up time of 18 months.^87^

Similarly, targeting LSCs has gained traction in AML, with the development of therapies designed to disrupt survival pathways in these cells. Venetoclax, a BCL-2 inhibitor, successfully activates apoptotic pathways in LSCs and offers a new therapeutic strategy for patients with high-risk or refractory AML^88,89^

In T-ALL, there are currently no therapies approved specifically for targeting the LSC population. However, several investigational therapeutics focus on modulating pathways critical to T-ALL LSC activity (Table 1).^17^ For example, Notch signaling, a central pathway in T-ALL development, has long been an attractive target.^104^ Early efforts involved γ-secretase inhibitors (GSIs), which block a critical step in Notch activation.^105^ The γ-secretase complex mediates a secondary cleavage of the Notch1 receptor to facilitate the release of intracellular Notch, which is subsequently translocated to the nucleus to activate transcription of Notch target genes.^106^ Several GSIs such as MK0752,^107^ PF-03084014^108^ and BMS-906024^109^ have undergone clinical trials in T-ALL patients.^17^ Other GSIs, including MRK-560,^91^ MRK-003,^90^ and DAPT^92^ have shown promise in pre-clinical studies, reducing LSC frequency and inhibiting leukemia engraftment in murine models. For instance, Habets et al. reported that inhibition of a subclass of γ-secretase complexes with MRK-560 significantly reduced leukemia burden in four PDX models *in vivo* .^91^ However, despite these advances, the translation of GSIs to standard clinical practice has been hindered by significant gastrointestinal toxicities.^110^

**Table 1.** Inhibitors targeting canonical self-renewal pathways in T-ALL.

Drug	Pathway	Specific effect	The model used in testing	Effect on LSC	Drug development stage*	Reference
γ-secretase inhibitor: MRK-003	Notch1	Notch pathway inhibition	*Tal1/Lmo2* transgenic mice model	Treatment reduced LSC frequency; all transplanted mice failed to develop the disease	Preclinical	^90^
γ-secretase inhibitor: MRK-560		Notch pathway inhibition	Leukemia mice model; Human T-ALL PDX mice	Treatment strongly mitigated leukemia development	Preclinical	^91^
γ-secretase inhibitor: DAPT		Notch pathway inhibition	Primary T-ALL cells; Human T-ALL PDX-mice	Lower T-ALL engraftment in mice transplanted with cells drug-treated	Preclinical	^92^
FDA-approved compounds and 2-ME2		MYC and SCL protein levels decrease	Thymocytes reprogrammed by the *SCL* and *LMO1* oncogenic transcription factors into self-renewing pre-LSCs; Transgenic mice model	2-ME2 abrogated pre-LSC viability in vitro and self-renewal activity in vivo	Preclinical	^93^
γ-secretase inhibitor: DAPT		*LYL1* and *LMO2* expression inhibition	Thymocytes reprogrammed by the *SCL* and *LMO1* oncogenic transcription factors into self-renewing pre-LSCs; Transgenic mice model	Lower T-ALL engraftment in mice transplanted with cells drug-treated	Preclinical	^94^
γ-secretase inhibitor: DAPT		Notch pathway inhibition	T cell lymphoma line: CUTLL1	Decrease of cell viability	Preclinical	^95^
Rapamycin, JQ1, and VX-680	MYC and PI3K-Akt	MYC and PI3K/Akt pathway inhibition	T-ALL cell lines: Jurkat, MOLT-3, MOLT-4, MOLT-16, CCRF-CEM; T cell lymphoma line: CUTLL1; *PTEN* null mice model	Treatment eliminated *PTEN* null LSCs	JQ1, and VX-680: Preclinical Rapamycin: Phase I, II (completed)	^96^
MK-2206	PI3K-Akt	Allosteric Akt inhibitor	T-ALL cell lines: MOLT-4, CCRF-CEM, CEM-R	Treatment induced apoptosis in a T-ALL patient cell subset (CD34þ/CD4/CD7), which is enriched in LSCs	Phase I, II (completed)	^97^
Rapamycin		mTOR complex 1 inhibition	*PTEN* null mice model	Long treatment of pre-leukemic *PTEN*-deleted mice blocked the formation of LSCs and prevented disease development	Phase I, II (completed)	^98^
BKM120		PIK3 inhibition	Primary T-ALL cells; T-ALL cell lines: Jurkat, RPMI-8402, BE-13, HPB-ALL, PF-382, P12-Ichikawa, DND-41, MOLT-4, CCRF-CEM, CEM-R	Treatment-induced apoptosis in the side population (SP) of cells that is enriched in LSCs	Phase I, II (completed)	^99^
JQ1	MYC	c-Myc pathway inhibition	*Tal1/Lmo2* transgenic mice; Primary mouse T-ALL cells; Primary human T-ALL cells	Treatment reduced LSC frequency	Preclinical	^100^
10058-F4, MYCi975	MYC-MAX	MYC–MAX complex formation	T-ALL cell lines: KOPT-K1 and Jurkat	Treatment induced cytotoxicity in T-ALL cells in a dose-dependent manner and down-regulated the expression of NOTCH1	Preclinical	^101^
PROTAC: ARV-825	Notch1-MYC-CD44	Downregulation of the Notch1-MYC-CD44 axis	T-ALL cell lines: CCRF-CEM, HPB-ALL, KOPT-K1, Loucy, MOLT4, SUP-T1; *PTEN* null mice model; Human T-ALL PDX-mice	Treatment reduced LSC frequency, induced cell cycle arrest and apoptosis; extended survival of mice engrafted with T-ALL from ARV-825 treated mice	Preclinical	^102^
Erlotinib	Wnt/β-catenin	Wnt/β-catenin inhibition	T-ALL cell lines: Jurkat; *6xTCF/LEF-miniP:sGFP* transgenic zebrafish; T-ALL CG1 zebrafish model	Treatment reduced self-renewal of T-ALL cell line, reduced LSC frequency and delayed disease formation in zebrafish models	Phase I, II (completed)	^67^
XAV-939		Wnt/β-catenin inhibition	T-ALL cell lines: HPBALL, RPMI8402; Human PDX cells	XAV-939 induced cytotoxicity in T-ALL cells in a dose-dependent manner	Preclinical	^103^

*Clinical studies focused specifically on leukemia.

Monoclonal antibodies targeting Notch, such as OMP-52M51, represent an alternative approach with fewer toxicities. Pre-clinical studies using PDX models demonstrated that treatment with the anti-Notch antibody significantly delayed the engraftment of T-ALL cells. This includes PDX derived from patients whose disease did not respond well to chemotherapy or who relapsed. For example, mice treated with anti-Notch1-treated mice were healthy or had low leukemia burden compared to control animals, suggesting decreased LSC function.^111^ Preliminary results from the phase I clinical trial on OMP-52M51 in patients with hematologic malignancies, including T-ALL, suggest a moderate efficacy and an acceptable safety profile, with diarrhea noted as the most common side effect.^112^

The PI3K/Akt/mTOR signaling pathway is also implicated in the self-renewal of LSCs in T-ALL.^13^ This pathway is negatively regulated by *PTEN*, a tumor suppressor gene altered in 11–27% of pediatric T-ALL cases.^113^ Therapeutic inhibition of this pathways has shown promise in targeting LSC-enriched populations. For instance, BKM120, a pan-PI3K inhibitor, preferentially induced apoptosis in the side population (SP) of cells enriched in LSCs, with a lesser effect on non-SP cells in primary T-ALL patient samples.^99^ Similarly, the Akt inhibitor MK-2206 induced apoptosis in a specific subset of cells (CD34+/CD4-/CD7-), which is enriched in LSCs,^97^ further demonstrating the potential of targeting this pathways.

In pre-clinical models, Guo et al. reported that the long-term treatment (3–4 months) of pre-leukemic *PTEN*-deleted mice with the mTOR inhibitor rapamycin blocked the LSC self-renewal and prevented disease development.^98^ Schubbert et al. demonstrated that treating mice engrafted with *PTEN*-null T-ALL cells with a combination of rapamycin and the MYC inhibitor JQ1 resulted in marked reduction of splenomegaly and significantly decreases in leukemia burden within seven days. Importantly, this combination treatment was found to significantly decrease the percent of Lin^−^;CD3^+^;c-kit^mid^ LSC-enriched subpopulation in the bone marrow.^96^

However, targeting the PI3K/Akt/mTOR pathway presents challenges due to its crosstalk with the Notch signaling pathway. Inhibition of PI3K/mTOR can inadvertently activate Notch signaling, which may lead to treatment resistance.^96,114^ This necessitates the need for combination therapies to simultaneously address interconnected signaling networks and effectively manage disease.

The NOTCH and PI3K/Akt/mTOR signaling pathways are also directly involved in regulating MYC expression, creating a synergistic network critical for leukemic progression. Indirect targeting of MYC has been vigorously investigated for decades.^115^ One promising strategy involves inhibiting MYC gene transcription, with JQ1 as a prominent example. JQ1 selectively targets the acetyl-lysine recognition motif of the BET family protein BRD4, leading to the suppression of MYC transcription and a reduction in leukemic stem cell frequency.^100^ Another approach targets MYC-MAX complex formation, which is essential for MYC-mediated transcriptional programs. Small molecule inhibitors 10,058-F4 and MYCi975 have demonstrated efficacy in disrupting MYC – MAX dimerization, resulting in suppressed growth of T-ALL cell lines.^101^ However, while these approaches show potential, relatively few compounds have been investigated in the context of LSCs in T-ALL, highlighting the need for further exploration in this area.

The Wnt signaling pathway is crucial for self-renewal in both normal^116^ and cancer stem cells^10^ and represents another significant therapeutic target in T-ALL. Inhibition of Wnt signaling in hematopoietic stem cells reduces their capacity to self-renew and regenerate the hematopoietic system.^117^ Dysregulated Wnt signaling is implicated in the malignant transformation and the maintenance of LSCs in AML and chronic myeloid leukemia (CML).^118,119^ In T-ALL, Giambra et al. observed activated Wnt signaling in a subpopulation of cells enriched for LSCs in NOTCH1-ΔE induced leukemia mouse model. Their findings showed an 85-fold decrease in LSC frequency following the transduction of leukemia cells with a dominant negative TCF (dnTCF) that inhibited β-catenin binding and prevented canonical activation of the pathway.

Pharmacological inhibition of Wnt signaling has shown pre-clinical efficacy. For example, XAV-939 demonstrated ability to inhibit the growth and proliferation of various T-ALL cell lines and human PDX cells *in vitro*.^103^ However, the role of Wnt signaling in normal tissue regeneration and hematopoiesis presents a significant challenge for clinical translation due to associated side effects.^120^ To overcome this challenge, our group utilized a zebrafish line that harbors a Wnt/β-catenin reporter (*6XTCF/LEF-miniP:dGFP)* to repurpose FDA-approved compounds with established safety profiles as Wnt/β-catenin inhibitors. Erlotinib emerged as a top hit and was found to inhibit the nuclear localization of β-catenin and reduce the expression of Wnt targets. Erlotinib inhibited the self-renewal of human T-ALL cells *in vitro* and reduced LSC frequency and delayed disease formation in zebrafish models.^67^

Despite significant progress in targeting T-ALL LSCs in cell cultures and pre-clinical models, clinical application of most of the inhibitors listed in Table 1 may be limited due to the dual role of these signaling pathways in both LSC maintenance and normal stem cell self-renewal. This overlap poses a major challenge, as therapeutic interventions often result in significant toxicity, making it difficult to exploit a therapeutic window. Identifying novel, LSC-specific targets that regulate self-renewal and survival is critical to advancing the translatability of research findings into clinical practice for T-ALL.

## 4. The mitochondria as a target organelle in T-ALL

Overcoming challenges associated with targeting traditional signaling pathways in T-ALL has prompted increased investigation into cancer cell metabolism, which has emerged as a promising area of research.^121^ Mitochondria are indispensable organelles responsible for energy production, metabolic regulation, and cell fate determination.^122^ In cancer, mitochondria are not only the hub of metabolic reprogramming but also key players in sustaining tumor survival, promoting proliferation, and mediating drug resistance. Traditionally, cancer cells have been characterized by their reliance on glucose for energy, a phenomenon known as the Warburg effect.^123^ However, increasing evidence describes the central role of mitochondria in regulating the metabolic switch between aerobic glycolysis and oxidative phosphorylation (OXPHOS), enabling the high rates of anabolism and bioenergetics necessary for oncogenic transformation.^124^

In addition to energy metabolism, mitochondria regulate apoptosis through calcium signaling. Tumors can modulate their sensitivity to apoptosis by altering calcium flux between mitochondria and the endoplasmic reticulum (ER) via proteins like the voltage-dependent anion channel (VDAC),^125^ disrupting this critical signaling axis. Mitochondria-generated reactive oxygen species (ROS) also play a pivotal role in cancer progression, activating survival and metastatic pathways, including nuclear factor−κB (NF-κB), PI3K/Akt, and the mitogen-activated protein kinase (MAPK)/extracellular-regulated kinase 1/2 (ERK1/2) pathways.^126,127^ Therapeutic strategies targeting mitochondrial ROS can overwhelm antioxidant defenses, destabilize mitochondrial membrane potential, and induce apoptosis.^128,129^

Drugs targeting mitochondrial metabolism have demonstrated success in leukemias such as AML, CML, and CLL, where they have overcome chemoresistance,^130–132^ induced apoptosis,^131^ and promoted cellular differentiation.^133^ Recent research in T-ALL has revealed similarly critical roles for mitochondrial function. T-ALL cell lines exhibit increased expression of OXPHOS genes compared to normal T cells and other cancer cell lines.^134^ A metabolism-focused CRISPR-Cas9 screen by Kong et al. identified mitochondrial complex I as essential for T-ALL cell survival, with loss of these genes proving lethal, especially in the presence of the mitochondria-targeted antioxidants like mito-vitamin E. Subsequently, the use of Phenformin, an inhibitor of mitochondrial complex I and OXPHOS, significantly reduced T-ALL burden in mouse models.^135^ These findings highlight the potential of targeting mitochondrial processes as a therapeutic strategy for T-ALL.

### 4.1. Mitocans as a new treatment strategy for T-ALL

With the established importance of mitochondrial pathways in T-ALL, the emergence of Mitocans – small molecule compounds that precisely target and destabilize mitochondrial functions – opens new avenues for effective treatment strategies.^136^ These compounds disrupt key mitochondrial processes, including mitochondrial ATP and DNA synthesis, the balance between OXPHOS and glycolysis, redox homeostasis, and mitochondrial-dependent apoptosis.^137^

Numerous mitochondrial targets in T-ALL have been identified and investigated for their therapeutic potential. Hexokinase, a key enzyme linking glycolysis and mitochondrial metabolism, is one such target, and its inhibition induces mitochondrial dysfunction and apoptosis in T-ALL cells.^138,139^ Pro-apoptotic Bcl2 family proteins, which regulate mitochondrial membrane permeability, are also critical targets, with studies showing their modulation can sensitize T-ALL cells to apoptosis.^140–145^ The mitochondrial voltage-dependent anion channel (VDAC) plays a role in maintaining mitochondrial integrity and regulating metabolite exchange, making it another promising target.^146^ Additionally, therapeutic approaches that destabilize mitochondrial redox homeostasis have been shown to increase ROS levels in T-ALL, leading to mitochondrial damage and cell death.^147–149^

Mitocans are effective through several mechanisms, including cytochrome c release,^150^ loss of mitochondrial membrane potential,^151^ disruption of the electron transport chain, and ultimately, the failure of ATP production.^139^ These effective collectively impair mitochondrial function and drive apoptosis, offering a powerful approach to overcoming drug resistance and eradicating leukemia cells. A comprehensive summary of these studies is provided in Table 2.

**Table 2.** Summary of mitochondrial-targeting agents tested in T-ALL.

Drug	Mitochondrial target/Vulnerability	The model used in testing	General Outcome	Drug development stage*	Reference
Poricoic acid A	↑ROS levels; ↓Complex I; ↓Cristae, and ↑mitochondria membrane density	T-ALL cell lines: Jurkat, MOLT-3, ALL-SIL, RPMI-8402	Autophagy, Ferroptosis	Preclinical	^152^
11- methoxytabersonine (11-MT)	↑ROS levels; ↓MMP; ↓PI3K/Akt/mTOR signaling pathway	T-ALL cell lines: MOLT-4, Jurkat, CCRF-CEM, CEM/C1	Apoptosis	Preclinical	^151^
Minocycline	↑ROS levels; ↓MMP; ↑*PINK1* and *PARKIN*	T-ALL cell line: Jurkat	Apoptosis	Preclinical	^153^
Cannabinoid CP55940	↑ROS levels; ↓MMP; ↑*PINK1* and *PARKIN*	T-ALL cell line: Jurkat	Apoptosis	Preclinical	^154^
WP1130	↓MMP; ↓*Mcl-1*	T-ALL cell lines: Jurkat, MOLT-4, HPB, CCRF-CEM	Apoptosis	Preclinical	^155^
Vorinostat/Quinacrine	↓MMP; ↓*Mcl-1*; ↓Bcl-2/Bax ratio; ↑*PARKIN*	T-ALL cell lines: Jurkat, MOLT-4; PDX mouse model	Apoptosis, Mitophagy blockage, and Tumor size decrease	Vorinostat: Phase I, II (completed and recruiting) Quinacrine or Combination of drugs: Preclinical	^140^
Cannabidiol	↓MMP; Cytochrome c release, ↑Ca^2+^,	T-ALL cell lines: MOLT-3, Jurkat, CCRF-CEM	Apoptosis	Preclinical	^156^
Tipifarnib	↓*SHMT2*, *MTHFD1* and *CTPS1*	25 T-ALL cell lines	Cytotoxicity	Phase I, II, III (completed)	^157^
Brusatol	↑ROS levels; ↓MMP	T-ALL cell lines: MOLT-4, CCRF-CEM	Cytotoxicity	Preclinical	^147^
Oxamate	LDH inhibition; ↑ROS levels; ↓MMP	T-ALL cell lines: Jurkat, DU528	Apoptosis	Preclinical	^158^
α-Pinene	↑ROS levels; ↓MMP; ↓ Basal and maximal OCR; ↑Glucose consumption and lactate production; ↑ EGR1-p53-Bax/Bcl-2-caspase cascade	T-ALL cell lines: EL-4, MOLT-44; PDX mouse model	Apoptosis, Tumor size decrease	Preclinical	^142^
Ferrocene derivatives	↑ROS levels; ↓MMP; ↓PI3K/Akt/mTOR signaling pathway	T-ALL cell lines: Jurkat, CEM-T4, RAJI, CA46, SNT8, SNK6	Apoptosis	Preclinical	^159^
SHIN2	Inhibition of mitochondrial isozyme *SHMT2*	T-ALL cell lines: MOLT-4, MOLT-3, Jurkat, KOPT-K1; PDX mouse model	Cytotoxicity, Sensitization to methotrexate, Tumor size decrease	Preclinical	^160^
PKHB1	↑ROS levels; ↓MMP; ↑ATP release	T-ALL cell lines: MOLT-4, CCRF-CEM, L5178Y-R	Apoptosis	Preclinical	^148^
ABT-199/Gemcitabine	↓MMP; ↓*Bcl2* and *Bcl-xL*;↑ *Bim* and *Puma*; Cytochrome c release, PARP cleavage	T-ALL cell lines: MOLT-4, Jurkat	Apoptosis	Venetoclax: Phase I, II, III (completed and recruiting) Phase IV (recruiting) Gencitabine: Phase I, II (completed and recruiting) Combination of drugs: Preclinical	^141^
MI-2	PARP cleavage; ↓Survivin, *Bcl-xL*, *Bcl-2* and *NF-ƙB*; ↑*Bax*	T-ALL cell lines: MOLT-4, CCRF-CEM	Apoptosis	Preclinical	^143^
Resveratrol, Quercetin, Genistein, Curcumin	↓MMP	T-ALL cell line: MOLT-4	Cytotoxicity	Preclinical	^161^
Melittin	↓OCR	T-ALL cell line: Jurkat	Cytotoxicity	Preclinical	^162^
Cannabidiol, Curcumin, Quercetin	↓MMP; ↑ROS levels; ↑Ca^2+^,	T-ALL cell line: Jurkat	Cytotoxicity	Preclinical	^163^
Cannabidiol, Tamoxifen	↓MMP; ↑Ca^2+^; Cytochrome c release	T-ALL cell lines: Jurkat, CCRF-CEM	Cytotoxicity	Preclinical	^150^
Xestospongin B	InsP3R inhibition resulting in a decrease of InsP3R-mediated Ca2+ transfer from the endoplasmic reticulum to mitochondria; ↓Basal and maximal OCR	T-ALL cell lines: Jurkat, CCRF-CEM	Cytotoxicity	Preclinical	^164^
Tamoxifen	↓MMP	T-ALL cell line: Jurkat	Apoptosis, Autophagy, Sensitization to dexamethasone	Preclinical	^165^
Apatinib and Chidamide	↓ basal and maximal OCR, ↓Enzymes involved in the citric acid cycle and oxidative phosphorylation, ↑Mitochondria-mediated apoptosis pathways	T-ALL cell lines: MOLT-4, Jurkat; PDX mouse model	Apoptosis, Tumor size decrease	Apatinib: Preclinical Chidamide: Phase I, II (recruiting) Combination of drugs: Preclinical	^166^
Decitabine	Mitochondrial morphology changes; ↓PI3K/Akt/mTOR signaling pathway	T-ALL cell line: MOLT-4	Apoptosis	Phase I, II and III (completed and recruiting)	^167^
Quercetin and Autophagy inhibitors	↓MMP; ↑ROS levels; ↓*BAG3*, *Mcl-1;*↑*Bak*, Caspase-9 -3 -8; PARP cleavage	T-ALL cell lines: Jurkat clones (J/Neo and J/BCL-XL)	Apoptosis	Preclinical	^144^
Ginsenoside 24-hydroxy-ginsengdiol	↓MMP; ↑ROS levels; ↑*Bax*, Caspase-9–3; Cytochrome c release	T-ALL cell lines: CCRF-CEM; PDX mouse model	Apoptosis, Tumor size decrease	Preclinical	^145^
Chidamide	↓MMP;↓ cFLI PL, HDAC 1 and HDAC 3	T-ALL cell lines: Jurkat, HUT-78	Apoptosis and Necroptosis	Phase I, II (recruiting)	^168^
Diacetyl hexamethylene diamine	↓MMP; ↓Bcl-2/Bax ratio; ↓Akt	T-ALL cell line: Jurkat	Apoptosis	Preclinical	^169^
TPEN and TPGS agents (T2 combo)	↓MMP; ↑caspase 3	T-ALL cell line: Jurkat	Cytotoxicity	Preclinical	^170^
VDAC1-derived peptides	Cytochrome c release, HK dissociation from mitochondria	T-ALL cell lines: MOLT-4, Jurkat	Cytotoxicity	Preclinical	^146^
ABT-737	↓MMP; ↑*Bax*, *Bid* and Caspase; Cytochrome c release;	T-ALL cell lines: MOLT-3, MOLT-4, CCRF-CEM, COG-VV-317	Apoptosis, Sensitization to chemotherapies	Preclinical	^171^
ABT-737	↓MMP; ↓*Bcl2* and *Mcl-1*; Cytochrome c release.	T-ALL cell line: CEM-c1	Apoptosis	Preclinical	^172^
ABT-263	↓Pro-survival Bcl2 family proteins	PDX mouse model	Tumor size decrease, Sensitization to chemotherapies	Phase I, II (completed)	^173^
Methyl jasmonate	↓ATP; Cytochrome c release; HK dissociation from mitochondria	T-ALL cell line: MOLT-4	Cytotoxicity	Preclinical	^139^
2-DG, LND, 3-BrPA	HK inhibition	T-ALL cell lines: MOLT-4, Jurkat	Sensitization to glucocorticoids	Preclinical	^138^
Resveratrol	↓MMP; ↑ROS levels	T-ALL cell line: Jurkat	Apoptosis	Preclinical	^174^
Arsenic trioxide	↑ROS; ↑*Bad*; ↓Akt	T-ALL cell lines: MOLT-4, Jurkat, CEM-C7, CEM-C1	Apoptosis	Phase I, II, III, IV (completed) Phase I, II (recruiting)	^149^
Vitamin K3, Vitamin C	↓MMP; ↑ROS levels; ↑*Nf-kB*, *p53*, c-Jun, Caspase-3	T-ALL cell line: Jurkat	Apoptosis	Preclinical	^175^
Tigecycline	↓Basal and maximal OCR; ↓ATP; ↑ROS levels	T-ALL cell lines: CCRF-CEM, DND-41, MOLT-4; PDX mouse model	Apoptosis, Sensitization to chemotherapies, Tumor size decrease	Phase I (completed)	^176^
Ara-C	↑ROS levels	T-ALL cell lines: MOLT-4, Jurkat	Apoptosis	Phase I, II, III, IV (completed and recruiting)	^177^

ROS- Reactive oxygen species, MMP- Mitochondria membrane potential, OCR- Oxygen consumption rate, HK- hexokinase, PDX- Patient-derived xenograft.

*Clinical studies focused specifically on leukemia.

## 5. The mitochondria as key driver of self-renewal in T-ALL

The extensive investigation of mitochondrial function across various cancers, including in T-ALL, has identified numerous mitochondrial targets that have shown anti-cancer activity in cell lines and animal models.^29,178^ This section focuses on advancing the understanding of the specific contribution of mitochondria to LSCs in T-ALL. While mitochondria are well established as a general target in T-ALL, the evolving understanding of metabolic heterogeneity in cancer underscores the importance of targeting mitochondrial vulnerabilities specific to distinct tumor subpopulations, such as cancer stem cells (CSCs) and LSCs.^179^

These subpopulations exhibit unique metabolic preferences influenced by several factors such as their driving mutations, cell of origin, epigenetic regulations, and tumor microenvironment.^180^ There is also increasing recognition of bioenergetic heterogeneity among CSCs. Specifically, CSCs of different tumors display different energy preferences (glycolytic vs. OXPHOS) depending on their spatial location, oxygen levels in the tissue, and prior exposure to chemotherapy.^181^

A large body of literature has evaluated the metabolic energy preferences for LSCs in AML.^182^ Proteomics analysis comparing LSCs with healthy age-matched hematopoietic stem and progenitor cells (HSPCs) revealed a shift from glycolysis to predominantly OXPHOS in LSCs.^183^ Various studies have targeted LSCs by inhibiting their OXPHOS metabolism. For instance, Lagadinou et al. demonstrated that when OXPHOS was inhibited by treatment with the mitochondrial inhibitors oligomycin and FCCP, LSCs failed to switch to glycolytic metabolism, indicating metabolic inflexibility, and then used ABT-263 to induce death in the LSC subpopulation.^78^ This finding was supported by Jones et al., who showed that LSCs utilize nutrients such as amino acids and fatty acids to fuel OXPHOS for energy production, and successfully targeted LSCs using a combination of venetoclax and azacytidine to inhibit both mitochondrial function and amino acid metabolism simultaneously.^184^ The investigation of mitochondrial function in self-renewal pathways in T-ALL offers a promising avenue for the development of LSC-specific targeted therapies similar to those seen in AML.

In T-ALL, maintaining a critical mitochondrial mass may be necessary for leukemia cells to mitigate the oxidative stress induced by chemotherapy. Burt et al. observed the transfer of mitochondria from mesenchymal stromal cells to T-ALL cells in a co-culture model via tunneling nanotube structures. Inhibiting these structures and thus depleting mitochondrial mass led to excessive ROS accumulation in T-ALL cells. Authors suggested that the protection of leukemia cells within a specific niche may also explain the failure of ROS-inducing therapies such as cytarabine (AraC) and daunorubicin to completely eradicate the disease in T-ALL patients.^185^

Expanding upon these insights into the importance of mitochondria in T-ALL, Zhong et al. investigated the role of the oxysterol-binding protein ORP4L in maintaining mitochondrial respiration in T-ALL by regulating calcium homeostasis. They found that primary T-ALL cells had increased ATP production rates, basal respiration, and ROS production compared to normal T cells. In addition, primary T-ALL cells and T-ALL cell lines demonstrated a reduced capacity to switch to aerobic glycolysis when treated with the mitochondrial inhibitor oligomycin. Short hairpin RNA (shRNA) targeting ORP4L reduced ATP production and disturbed the calcium mitochondrial signaling axis, resulting in low engraftment of primary T-ALL cells in mice, indicative of impaired self-renewal capabilities.^186^ Subsequently, ORP4L was shown to be selectively expressed on LSCs in AM, where knockdown of ORP4L resulted in decreased colony formation and energy production.^187^ Most recently, it was discovered that ORP4L deletion can prevent the induction of T-cell leukemogenesis by human T-cell leukemia virus 1.^188^ Together, these findings suggest a central role of mitochondrial metabolism and its regulators in the development and maintenance of T-ALL.

Another key facet of mitochondrial function explored in T-ALL is OXPHOS. Baran et al. described a functional link between activating *NOTCH1* mutations in T-ALL and OXPHOS. Bioinformatics analysis of T-ALL data sets indicated the Notch-1 bound target genes were predominantly associated with the OXPHOS pathway and mitochondrial electron transport chain. This connection was biologically validated using pre-LSCs from the murine *SCL-LMO1* transgenic model, where activation of Notch transformed these cells into hypercompetitive leukemia-propagating cells, leading to aggressive T-ALL without any latency. Moreover, in the presence of the activating Notch1 ligand DL4, inhibition of OXPHOS with the small molecule IACS-010759 effectively suppressed T-ALL development in a dose-dependent manner. Notably, cells were insensitive to treatment in the absence of the ligand, further linking Notch activation to OXPHOS in T-ALL. IACS-010759 also significantly reduced the basal and maximal oxygen consumption rates, as well as ATP production, across 11 different T-ALL cell lines, with a higher statistical significance in NOTCH-mutant cells compared to normal T lymphocytes. The bioenergetic changes correlated with decreased viability in T-ALL cell lines, patient-derived xenografts, and patient-derived primary samples. These findings emphasize the critical role of mitochondrial metabolism in the activation of pre-LSCs and the maintenance of disease in the context of activated Notch, one of the most prevalent driver mutations in T-ALL.^134^

Conversely, other studies have indicated a different mitochondrial adaptation in T-ALL, where there is a shift toward glycolysis for maintaining self-renewal. Fahy et al. examined T-ALL cells within the context of the bone marrow microenvironment to explore mechanisms of therapy resistance. LSCs are thought to predominantly reside in the hypoxic bone marrow niche.^103^ Under hypoxic conditions designed to mimic the bone marrow microenvironment, T-ALL cells entered a state of growth arrest, or dormancy, and exhibited increased chemotherapy resistance. These traits are characteristic of the LSC phenotype. When exposed to low oxygen levels, primary T-ALL cells had reduced retention of the MitoTracker stain, indicating reduced mitochondrial content, and increased levels of mitochondrial depolarization, indicative of decreased mitochondrial activity. In addition, elevated lactate levels in the cell culture media suggested a shift toward anaerobic glycolysis. These data suggest a significant metabolic rewiring of mitochondria in response to hypoxic conditions, such as may be found in the LSC bone marrow niche.^189^

Building on these insights into metabolic adaptation, Gachet et al. examined genetic factors contributing to poor prognosis in T-ALL associated with chromosome 6q deletion and found a ribosomal-mitochondrial axis involved in leukemogenesis. Their integrated genomic approaches identified *SYNCRIP* and *SNHG5* as candidate haploinsufficient genes. Silencing these genes significantly accelerated the *Tal1/Lmo1/Notch1-*induced T-ALL development in mice. CRISPR/Cas9 mediated deletion of those two genes in the CCRF-CEM human T-ALL cell line led to global downregulation of ribosomal pathways and oxidative phosphorylation. Clones lacking these genes had reduced mitochondrial respiration and enhanced glycolysis compared to non-edited controls, indicative of profound metabolic shifts. Moreover, to directly assess the impact of reduced OXPHOS on leukemogenesis, PDX-derived human T-ALL cells were treated with tigecycline, an inhibitor of mitochondrial protein translation, followed by an *in vitro* limiting dilution assay and immunotyping. Tigecycline treatment induced a shift toward the greater CD34 and lower CD8 expression indicative of increased clonogenicity.^190^ These findings highlight the complex interplay between genetic mutations and alterations in the cell’s energy metabolism, in this case through the ribosomal-mitochondrial axis, that impact the self-renewal properties of the LSC.

While the role of mitochondrial metabolism in T-ALL self-renewal is increasingly recognized, there remains a lack of consensus on the specific contributions of mitochondria, as evidenced by the conflicting findings summarized in Table 3. One potential explanation for these discrepancies is that many findings related to altered mitochondrial metabolism were byproducts of research focused on another primary target rather than from direct interrogation of mitochondrial functions in T-ALL self-renewal. An unbiased, systematic investigation of key targets known to regulate mitochondrial processes, followed by functional assays of self-renewal, could conclusively link mitochondrial function to LSC frequency or other indicators of self-renewal. Additionally, metabolic heterogeneity within different T-ALL models may contribute to inconsistent findings, leading to diverse interpretations of metabolic alterations. Ultimately, addressing these discrepancies through comprehensive and targeted research could lead to significant advancements in mitochondrial-focused therapies in T-ALL, enhancing treatment strategies for this challenging leukemia subtype.

**Table 3.** Mitochondrial vulnerabilities that affect self-renewal in T-ALL.

Mitochondrial Vulnerability	Mechanism of Action in Mitochondria	Models Used for Testing	General Outcome	Drug(s) Used	Reference
Mitochondrial respiration	ALL EVs were enriched with cholesterol, which accelerated the mitochondrial metabolism and the loss of quiescence in targeted healthy HSPC	PDX mouse model	ALL cells produced EVs that target endogenous murine HSPC in bone marrow disturbing their quiescence and maintenance	None	^191^
Mitochondrial calcium concentration	*ORP4L* expression in T-ALL cells mediates G protein-dependent signaling and leads to translocation and activation of PLCβ3 to maintain calcium homeostasis and bioenergetics	Primary T-ALL cells and PDX mouse model	*ORP4L* supports mitochondrial oxidative phosphorylation for T-ALL cell survival	None	^186^
Mitochondrial respiration	Deletion of chromosome 6q results in downregulation of genes *SYNCRIP* and *SNHG5* involved in mitochondrial function. Also, it reduced basal and maximal mitochondrial respiration	Primary T-ALL cells; T-ALL cell line: CCRF-CEM; T-ALL mouse model; PDX mouse model	Deletion of chromosome 6q inactivated *SYNCRIP* and *SNHG5* genes results in metabolic re-wiring and increased leukemia-initiating activity	Tigecycline	^190^
Mitochondrial content and membrane potential	Hypoxia-induced low mitochondrial mass and depolarization of the mitochondria membrane	Primary T-ALL cells; PDX mouse model	Hypoxia-induced *HIF-1α* expression and slowed down T-ALL growth through increased quiescence of leukemic cells, inducing drug resistance	Sensitization to chemotherapies	^189^
Mitochondrial respiration	IACS-010759, an inhibitor of mitochondrial complex I, disrupts oxidative phosphorylation and redox balance in *NOTCH1*-mutated T-ALL and pre-leukemic cells	T-ALL cell lines: Jurkat, PF-382, 1301, TALL-1, Loucy, P12-Ichikawa, MOLT-3, MOLT-4, CCRF-CEM, SUPT1 and KOPT-K1; T-ALL primary cells; PDX mouse model	OXPHOS downstream of Notch1 is essential for preleukemic and leukemic stem cell function. OXPHOS pathway blockade induced profound metabolic shutdown in T-ALL cells with *NOTCH1* mutations, in turn resulting in apoptosis	IACS-010759	^134^

EVs- Extracellular Vesicles, PDX- Patient-derived xenograft, HSPC- Hematopoietic Stem and Progenitor Cells, OXPHOS- Oxidative phosphorylation

## 6. Conclusion

Poor post-relapse outcomes remain a significant challenge in treating T-ALL. Targeting leukemia stem cells holds considerable promise for eliminating relapse and improving patient outcomes. However, targeting the canonical signaling pathways central to LSC function, such as Notch, Akt, and Wnt, is complicated by dose-limiting side effects due to their importance in normal tissue development and homeostasis.

Metabolic heterogeneity within tumors is increasingly recognized, with cancer stem cells, including LSCs, often exhibiting metabolic phenotypes distinct from the bulk tumor. This highlights the potential of targeting metabolic pathways to specifically inhibit self-renewal and LSC function. In this review, we analyzed the role of the mitochondria in the context of T-ALL self-renewal. Although Mitocans are widely used as a general T-ALL target, few studies detail the specific contributions of mitochondria to T-ALL LSC maintenance and behavior. This gap in knowledge can be partly explained by the current lack of robust surface markers for LSCs in T-ALL and the need for functional assays to accurately assess LSC enrichment.

Moving forward, there is an opportunity to systemically dissect the role of various mitochondrial processes in T-ALL self-renewal. Such investigations could provide promising avenues for therapy development, leading to the identification of targeted agents that specifically disrupt self-renewal in T-ALL and possibly other cancer stem cells with similar metabolic dependencies.
